# Geographical and Temporal Analysis of Tweets Related to COVID-19 and Cardiovascular Disease in the US

**DOI:** 10.1080/19475683.2022.2133167

**Published:** 2022-10-21

**Authors:** Xuan Zhang, Lan Mu, Donglan Zhang, Yuping Mao, Lu Shi, Janani Rajbhandari-Thapa, Zhuo Chen, Yan Li, José A. Pagán

**Affiliations:** a Department of Geography, University of Georgia, Athens, GA, USA; b Division of Health Services Research, Department of Foundations of Medicine, New York University Long Island School of Medicine, Mineola, NY, USA; c Department of Communication Studies, California State University Long Beach, Long Beach, CA, USA; d Department of Public Health Sciences, Clemson University, Clemson, SC, USA; e Department of Health Policy and Management, College of Public Health, University of Georgia, Athens, GA, USA; f Department of Population Health Science and Policy, Icahn School of Medicine at Mount Sinai, New York, NY, USA; g Department of Public Health Policy and Management, School of Global Public Health, New York University, New York, NY, USA

**Keywords:** Coronavirus disease 2019 (COVID-19), cardiovascular disease (CVD), spatial and temporal analysis, Twitter, geotag proxy

## Abstract

The COVID-19 pandemic has resulted in more than 600 million confirmed cases worldwide since December 2021. Cardiovascular disease (CVD) is both a risk factor for COVID-19 mortality and a complication that many COVID-19 patients develop. This study uses Twitter data to identify the spatiotemporal patterns and correlation of related tweets with daily COVID-19 cases and deaths at the national, regional, and state levels. We collected tweets mentioning both COVID-19 and CVD-related words from February to July 2020 (Eastern Time) and geocoded the tweets to the state level using GIScience techniques. We further proposed and validated that the Twitter user registration state can be a feasible proxy of geotags. We applied geographical and temporal analysis to investigate where and when people talked about COVID-19 and CVD. Our results indicated that the trend of COVID-19 and CVD-related tweets is correlated to the trend of COVID-19, especially the daily deaths. These social media messages revealed widespread recognition of CVD’s important role in the COVID-19 pandemic, even before the medical community started to develop consensus and theory supports about CVD aspects of COVID-19. The second wave of the pandemic caused another rise in the related tweets but not as much as the first one, as tweet frequency increased from February to April, decreased till June, and bounced back in July. At the regional level, four regions (Northeast, Midwest, North, and West) had the same trend of related tweets compared to the country as a whole. However, only the Northeast region had a high correlation (0.8–0.9) between the tweet count, new cases, and new deaths. For the second wave of confirmed new cases, the major contributing regions, South and West, did not ripple as many related tweets as the first wave. Our understanding is that the early news attracted more attention and discussion all over the U.S. in the first wave, even though some regions were not impacted as much as the Northeast at that time. The study can be expanded to more geographic and temporal scales, and with more physical and socioeconomic variables, with better data acquisition in the future.

## 1. Introduction

Cardiovascular disease (CVD) is the leading cause of mortality globally (Virani et al. 2020), and in the United States (U.S.), it is the underlying cause of more than one-third of total deaths in the country (Ali and Newman 2009). Clinical data indicate a high prevalence of pre-existing CVD among COVID-19 patients (Clerkin et al. 2020; Wang et al. 2020; Nishiga et al. 2020). In addition, COVID-19 susceptibility is strongly related to CVD (Clerkin et al. 2020; Zheng et al. 2020).

Twitter is a popular online social media platform allowing users to tweet and interact with short messages, images, and videos in real-time. Statistics show that one-third of people with a social media profile use Twitter (Hu et al. 2020; Gohil, Vuik, and Darzi 2018), and as of January 2021, Twitter has 353 million active users (Kemp 2021). The massive volume of tweets makes it a handy source to analyse people’s opinions, sentiments, and mobility (Mackey et al. 2017; Jurdak et al. 2015; Tumasjan et al. 2010).

Researchers have applied Twitter data in various health research for topic analysis, surveillance, engagement, intervention, and more (Sinnenberg et al. 2017). For example, Francis (2019) analysed the conversations and resonance triggered by a disclosure tweet about an artist’s mental suffering. Researchers have developed a disease surveillance system and monitored real-time flu and cancer tweets (Lee, Agrawal, and Choudhary 2013). Upon that, some scholars looked at the tweets with location tags, or geotags, to tackle health-related questions. For instance, geotagged tweets were used to understand the effect of climate and seasonality on depression (Yang, Mu, and Shen 2015) or monitor seasonal influenza’s spatial and temporal aspects (Tsou et al. 2013). Tweets with location information can be insightful to reveal not only the temporal aspect of our chosen topic but also the geographical perspective of the phenomena.

This study aims to identify the spatiotemporal patterns and correlations of CVD and COVID-19 related tweets with COVID-19 daily cases and deaths at the national, regional, and state levels. We proposed and validated the feasibility of using Twitter users’ registration state as a proxy of tweeting location, especially when geotagged tweets are limited. We analysed the geographical and temporal aspects embedded in the related tweets during the early stage of this pandemic.

## 2. Methods

### 2.1 Study area

The U.S. has the largest number of Twitter users (62.55 million) (Clement 2020). When our data collection ended on August 1, 2020, the U.S. had more than a quarter of confirmed COVID-19 cases and more than one-fifth of deaths worldwide, although its population was only 4.25% of the world population (World Health Organization 2021). There were intensive debates in the U.S. on the mandate of masks and face coverings, whether COVID-19 is real or a hoax, and shelter-in-place orders, among others, right before the 2020 presidential election (Woolfolk 2020; Chen et al. 2021). All the information influenced people’s perceptions and responses to COVID-19.

### 2.2 Data collection

We applied for and received approval to collect historical full-archive data to use the Twitter premium application programming interface (API), allowing users to query full-archive tweets since 2006 (Twitter Inc 2020a). We used Python code to collect original English tweets mentioning both one of the COVID-related keywords and one of the CVD-related keywords (Table 1) from February 1 to August 1 2020 (Coordinated Universal Time or UTC) using Search Tweets API (Twitter Inc 2020b). The CVD keywords were generated from the top five cardiac diagnoses on ICU admission (Wunsch et al. 2011). Although the number of retweets can show the impact of the original tweets, we only collected original tweets, which provide a more personal understanding and interpretation of this topic. The original tweets include not only the initial tweets posted by a user but also quotes (tweets quoting existing tweets) and replies. We limited the language to English to ensure a consistent language and context environment for data collection and processing. We did not specify the geographic extent when collecting data but zoomed into the U.S. for temporal, geographical, and content analyses. In total, we collected 131,674 original tweets satisfying all the above criteria over six months.

### 2.3 Temporal and geographical data analyses

**Figure 1.**
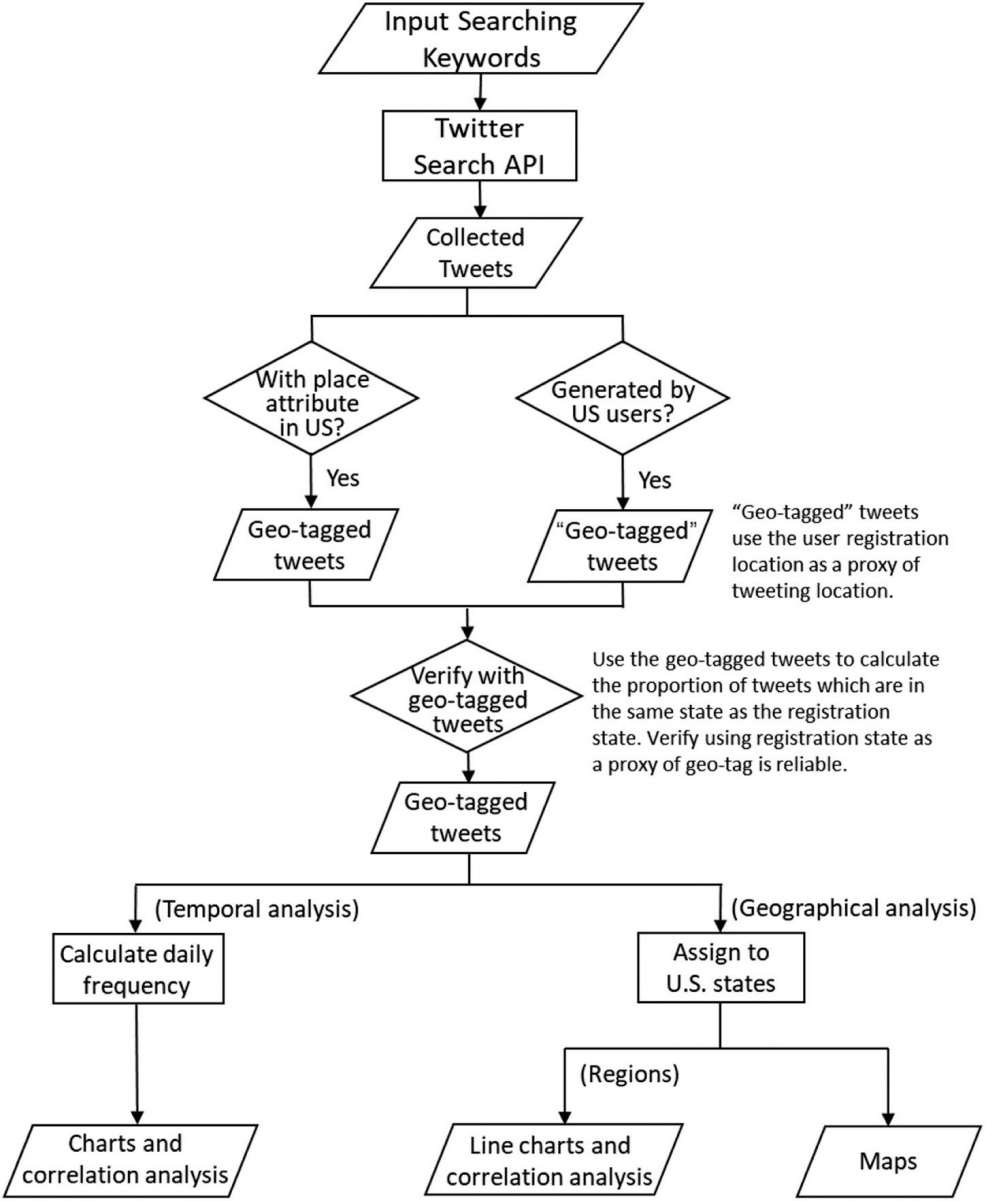
Research design.

We processed the collected tweets for temporal and geographical analyses. The general research design is shown in Figure 1. We converted the data from UTC (until August 1) to Eastern Time (E.T.) to fit the U.S. timeframe better. After the conversion, the data had full coverage from February 1 to July 31 E.T. In general, only a tiny portion (1–2%) of the tweets are geotagged (Twitter Developer 2020). Since the pandemic, people’s travel plans and activity spaces have changed dramatically due to the social distancing requirement and shelter-in-place orders (Schaper 2020). The U.S. Transportation Security Administration reported significantly fewer travellers through airports in 2020, with about a 50% decrease in March and a 95% decrease in early April (Nguyen and Animashaun 2020), compared with the previous year. Knowing this background information, we assumed that people were still in the same state as they registered for Twitter accounts and further justified the situation as follows. Each tweet is associated with a user, which may provide location information when he/she registers. In our data, there were 38,932 tweets generated by users whose profiles indicated living in the U.S. Among them, 1,172 tweets had meaningful geotags (excluding geotags such as poolside). We compared the state information from the geotags with that from users’ profiles and found that 89.68% of the users matched their registration state with the geotagged state. The percentage exceeded 90% if we consider Washington D.C. as part of Virginia or Maryland, as people who work in D.C. may live in the nearby states.

Additionally, only 2.3%-2.4% of Americans moved out of state yearly based on the 2010–2020 5-year American Community Survey data (U.S. Census Bureau, 2022). Due to the two reasons of sample geotag checking and the migration rate, we used the registration state as a proxy for the location information of the tweet during our study time. We extended the geotagged tweet group using the registration state as the tweeting location. The following analyses were based on the tweets with geotags in the U.S. or generated by users registered in the U.S.

The goal of the temporal analysis is to track the volume changes of tweets mentioning the related terms over time and evaluate them as the pandemic evolved. We plotted the histogram of daily tweets. We also downloaded and processed data on COVID-19 for the newly confirmed cases and deaths in the U.S. (Ritchie et al. 2020) and observed weekly oscillations between weekdays and weekends. Next, we followed the general method of the 7-day moving average (Centers for Disease Control and Prevention 2020; World Health Organization 2021) to obtain the smoothed value. For the smoothed values, we also calculated Spearman’s rank correlation coefficient between tweet count, newly confirmed cases (new cases), and newly confirmed deaths (new deaths). Spearman correlation measures the non-linear association between two variables (Lehman 2005). In the range of −1 to 1, the correlation coefficient’s sign indicates the direction of the association, and higher value means observations have a higher correlation between the two variables. Then we used the min-max normalization on the smoothed tweet count, new cases, and new deaths to transfer them into 0–1 scale, and visualized the temporal trends.

After further assigning tweets to the state or state-equivalent level and excluding the tweets that cannot be assigned, we visualized the monthly tweet count by the state to see the geographical trend. We also created a map with each state’s tweet count per 100,000 people to net out the population influence.

We applied the same methods at the regional level using the same set of variables – tweet count, new cases, and new deaths for four regions (Midwest, Northeast, West, and South) (U.S. Census Bureau 2021). We conducted 7-day average smoothing, Spearman’s rank correlation coefficients, rate by population, min-max normalization, and visualization.

## 3. Results

We collected 131,674 original tweets mentioning both COVID-19 and CVD-related keywords by 102,206 unique users worldwide. Only 2,741 (2.08%) of the collection were geotagged. Among them, users of 1,172 geotagged tweets had their registration location in the U.S. After justifying that about 90% of geotags were the same as the states that users registered, we used the registration state as a proxy of the tweeting location. Of all collected tweets, 44,967 (34.15%) are either geotagged in the U.S. or generated by a user registered in the U.S. However, users would be assigned to the U.S. centroid location in Kansas if they did not provide a more specific location other than the country in registration. We excluded tweets assigned to the U.S. centroid and those whose geotags cannot locate a state. We consider the remaining tweets to be assigned to a state based on either geotag or user registration information as geo-specific tweets. While analysing the data, we noticed some users intended to have exactly the same tweet but mentioned (@) different Twitter users over 400 times a day. We removed the highly duplicated tweets generated by the same user on the same day to keep the unique tweet that provided new information. The following results are based on the 34,610 geo-specific tweets with geotags in the U.S. or generated by U.S. users. To check the false positives in the data, we sampled 1% of tweets and manually reviewed them. Out of 350 tweets, only one (0.3%) is unrelated to COVID-19 and CVD. The tweet is about George Floyd and shared a news article about George Floyd, COVID-19, and CVD.

**Figure 2.**
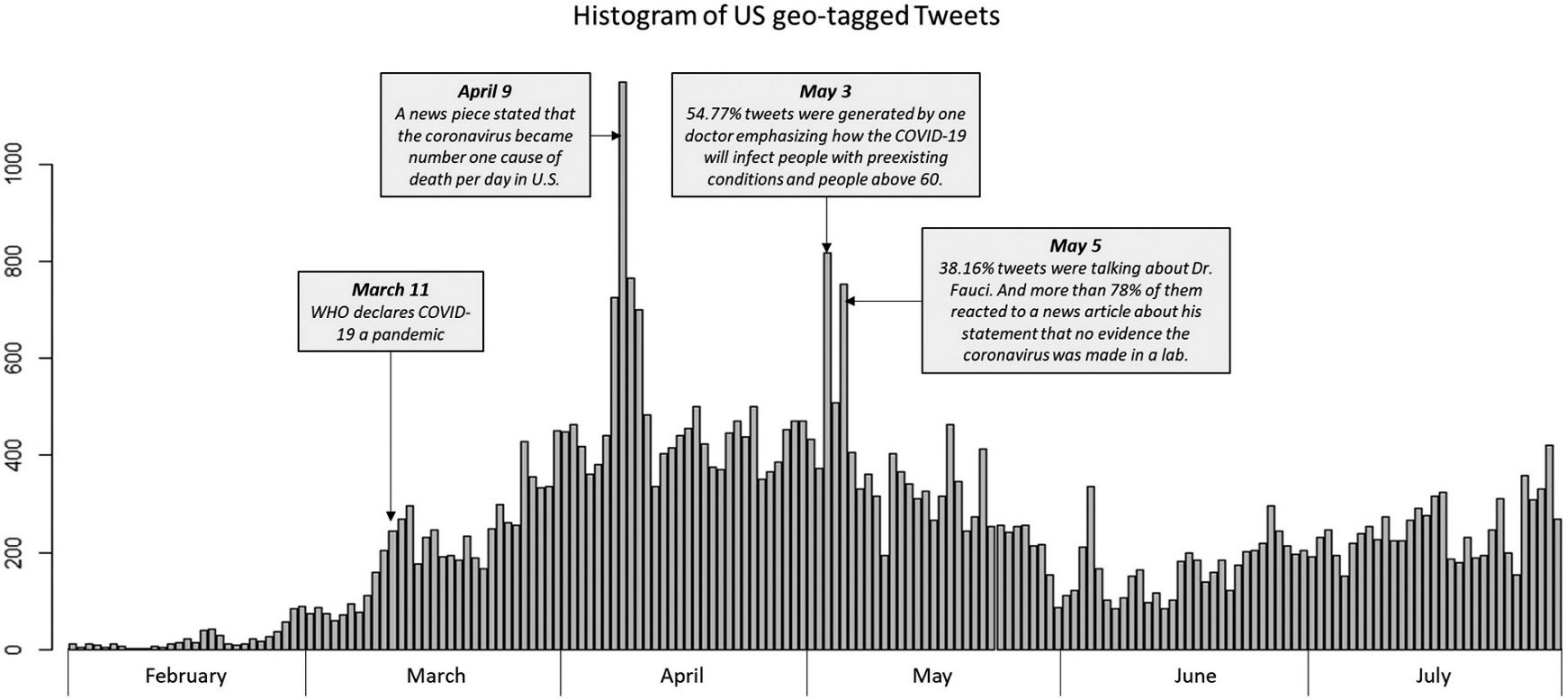
Histogram of geotagged tweets in the U.S.

The temporal trend of tweet frequency (before removing duplicate tweets) is shown in Figure 2. We can see a noticeable increase around March 11 when WHO declared COVID-19 a global pandemic (WHO 2020). The overall peak during the six months was on April 9 when a news piece came out and caught much attention by stating that COVID-19 became the number one cause of death per day in the U.S., surpassing heart disease and cancer (Impelli 2020). Another peak was around early May. On May 3, a doctor posted 448 tweets (54.77% of the tweets we collected that day), emphasizing how COVID-19 would infect people with pre-existing conditions, such as heart disease, and people over 60 years old, while young people will likely not have severe symptoms. On May 5, more than one-third of the tweets were about Dr. Fauci. Among them, more than 78% talked about his statement that coronavirus was not made in a lab (Akpan and Jaggard 2020).

After removing highly duplicated tweets, we applied the 7-day moving average method and smoothed tweets, new cases, and new deaths. We calculated Spearman’s rank correlation coefficient between them. Table 2 indicated a high correlation (coefficient = 0.88) between smoothed tweet count and new deaths. The coefficients were more than 0.60 between smoothed tweet count and new cases, and between smoothed new cases and new deaths.

**Figure 3.**
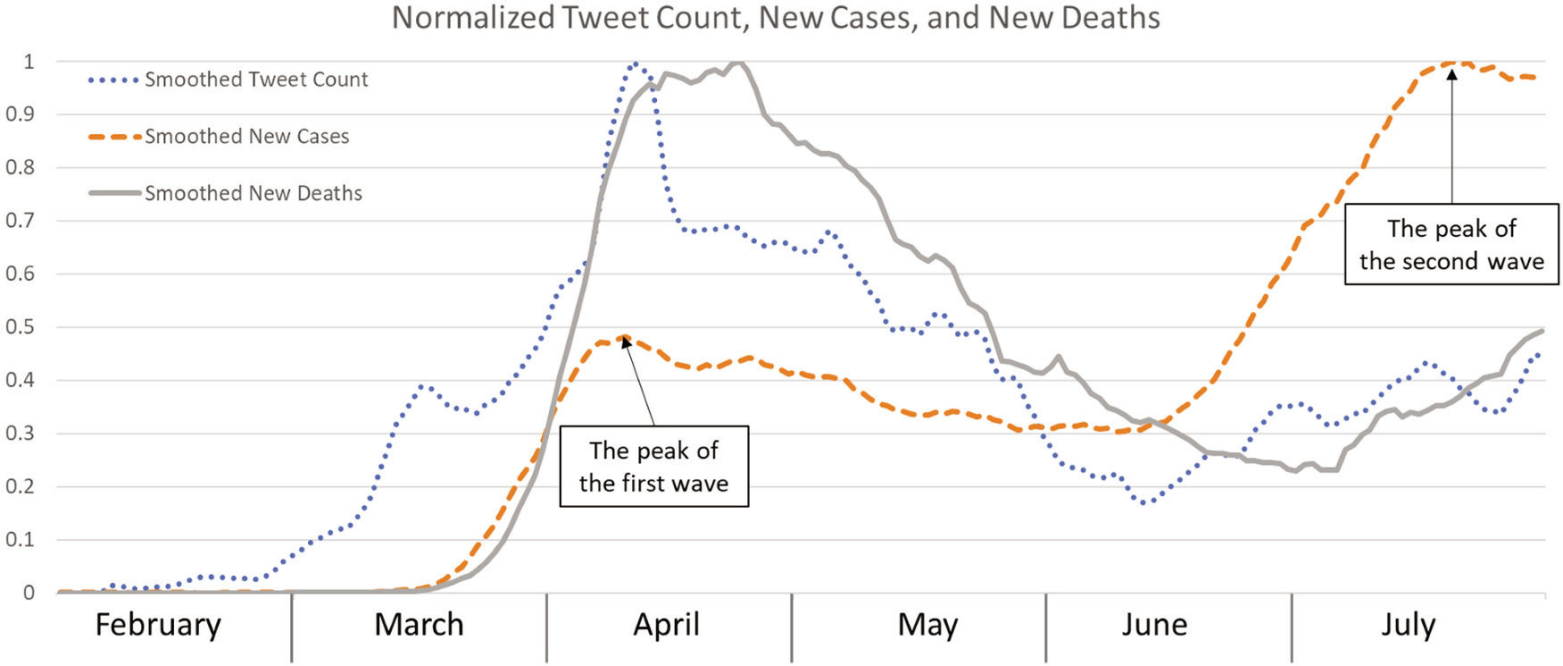
Normalized tweet count, new cases, and new deaths of COVID-19 in 2020.

Figure 3 shows that the normalized (value range: 0–1) tweet count (dotted line) and new cases (dashed line) have similar and increasing trends from February to mid-April to reach the peak and a decreasing trend till mid-June before the second wave of new cases and tweeting. However, reflected by the tweet count, the second increase of new cases from mid-June to mid-July did not ripple much in the Twitter community, reflecting the pandemic fatigue phenomenon. During the six months, the temporal pattern of tweet count synchronized more with the change in the death toll (solid line).

**Figure 4.**
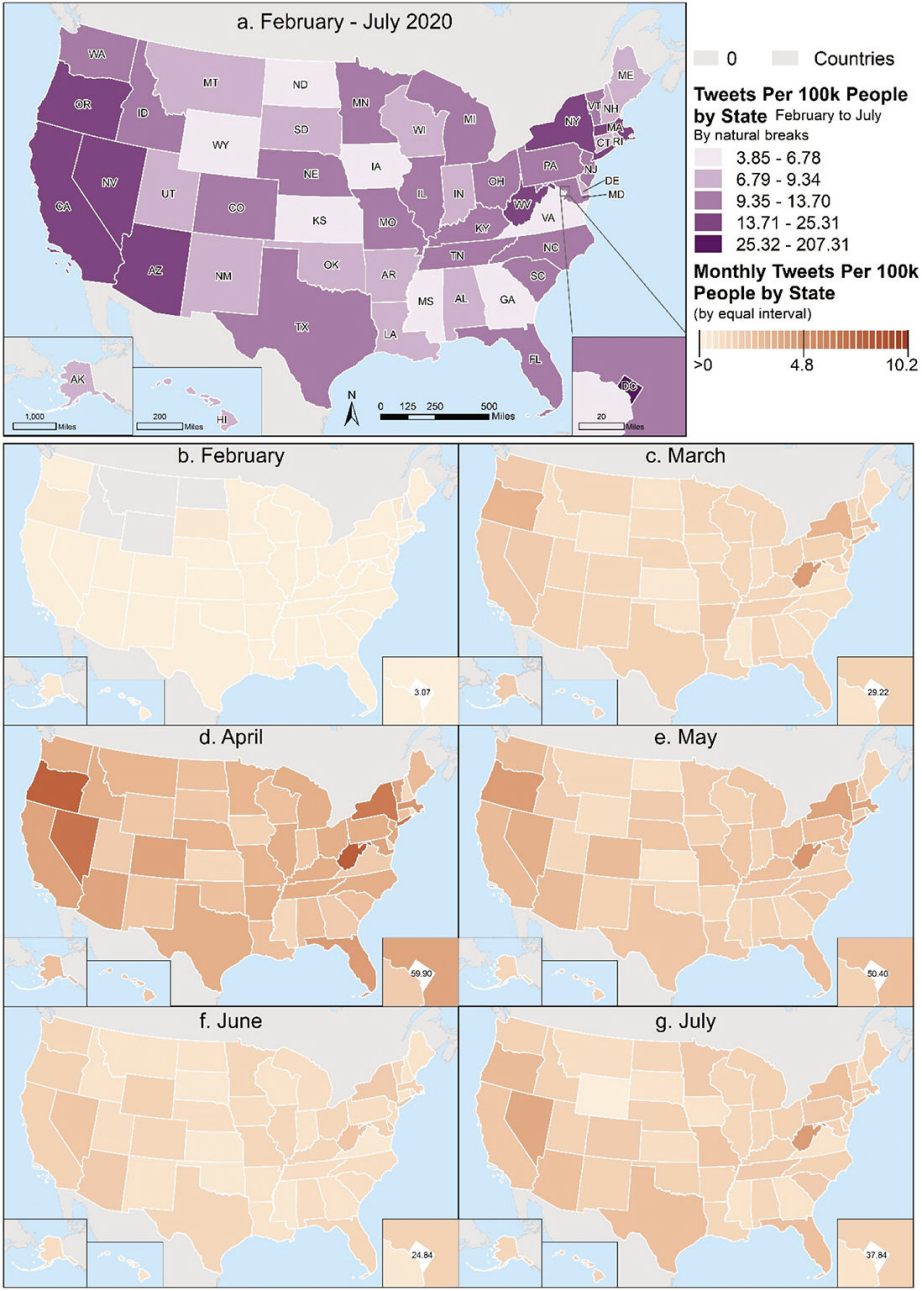
Geotagged tweets per 100,000 population by state (in Total and by Month).

Geographically, we visualized tweet count per 100,000 population (Figure 4) at the state level. West coast states, New York, Massachusetts, West Virginia, and Washington DC, stand out for the entire data collection period (Figure 4a). From February to April, there is a clear trend that more states were having related geotagged tweets and with increasing frequency. Then the intensity decreased from April to June and bounced back with more tweets in July.

**Figure 5.**
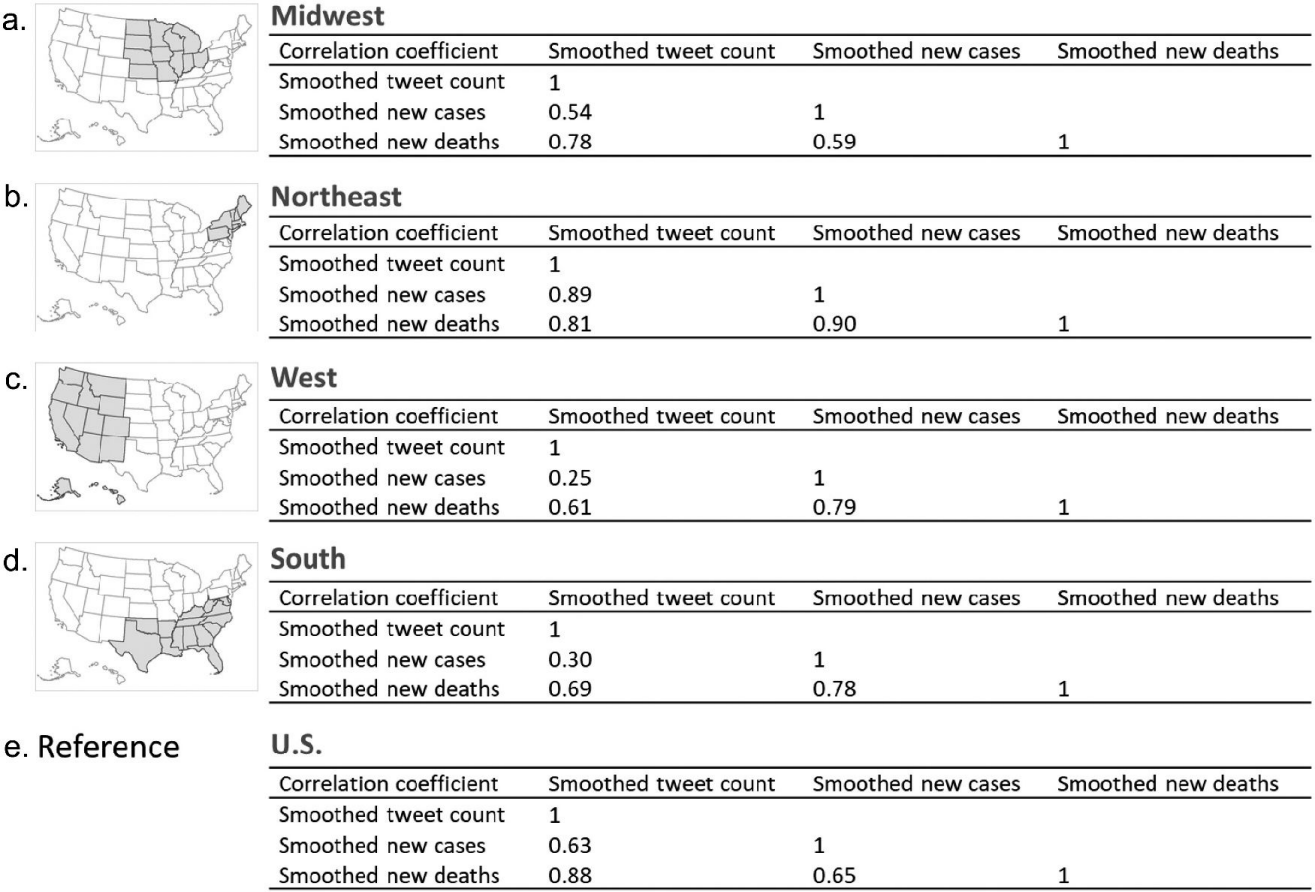
Correlation coefficients between smoothed tweet count, new cases, and new deaths of COVID-19 across regions.

Regionally, the correlation coefficient varies between variables (Figure 5). Generally, the regional coefficient of tweets and deaths (0.61–0.81) were all lower than the national value of 0.88 (Table 2 or Figure 5e). The coefficient between the tweet count and new cases ranges from 0.25 (West) to 0.89 (Northeast), compared to the national value of 0.63. For the coefficient between new cases and deaths, all regions have higher values (0.78–0.90) than the national value (0.65), except for Midwest (0.59). The Northeast region had a high correlation (0.81–0.9) between all three pairs of variables.

**Figure 6.**
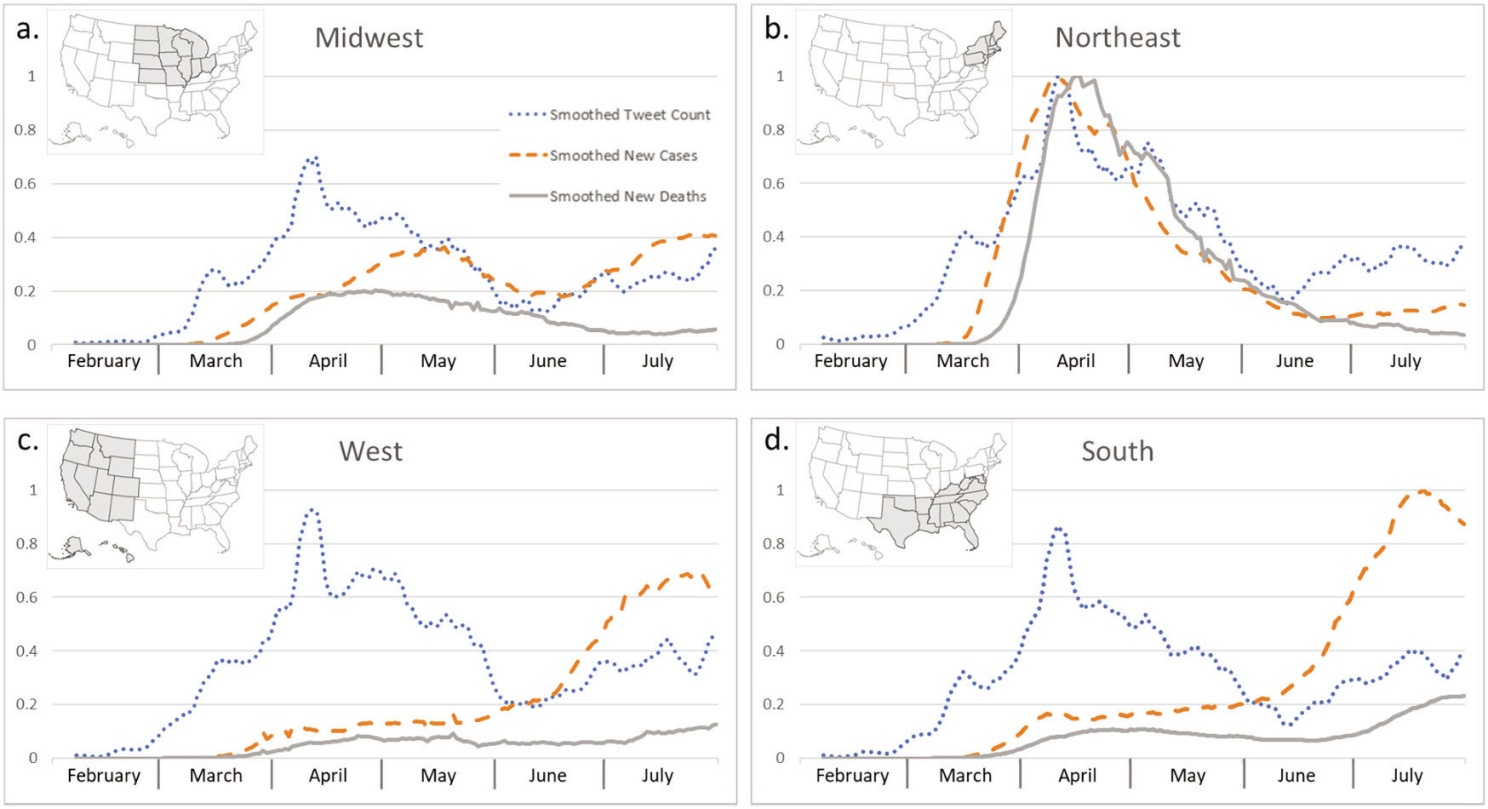
Line charts of normalized tweet count, new cases, and new deaths of COVID-19 across regions.

By plotting the normalized variables (Figure 6), we see similar trends of smoothed tweet count (dotted line) across four regions. The Northeast had a leading magnitude over the West, South, and Midwest. For the new cases (dashed line), the Northeast had its peak in early April and a decreasing trend starting in May. Comparing that, the South and West had their peak time in July, and the magnitude of the South is comparable to the Northeast’s April peak. Midwest had a much smaller magnitude than the other regions but with two humps in May and July. In terms of new cases and deaths (solid line), the Northeast was hit the hardest and earliest in April. While the engagement of tweeting (magnitude and trend of smoothed tweet count) was similar for the Northeast and West, the trend and magnitude of the case and death were quite different.

## 4. Discussion

Twitter is a real-time platform where people or organizations share their opinions, advice, and facts to reach a broader audience. This study presents an analysis of COVID-19 and CVD tweets during the early stage of the pandemic (February 1 to 1 August 2020) when we faced an unknown new virus that spread fast and dramatically influenced our lives. We used users’ registration location as a proxy of tweeting location to enlarge our dataset of geotagged tweets at the state level and analysed the geographical and temporal intensity of COVID-19 and CVD-related tweets. According to our analysis, the trend of related tweets is highly similar to that of COVID-19 new cases and deaths of the first wave in the U.S. The second wave caused another rise in the related tweet but not as much as the first one, and statistically, the tweet count has a higher correlation coefficient (0.88) with the daily death toll than the new cases. Regionally, they had different magnitudes and trends for tweet count, cases, and deaths. While all regions had similar trends of tweet counts for the country, only the Northeast region had a high correlation (0.81–0.9) between all three variables (tweet count, new cases, and new deaths). This could be related to the fact that the Northeast region, especially New York City, was the epicentre of the COVID-19 outbreak in the U.S. during the Spring of 2020 (Bialek et al. 2020) and the rising cases and deaths led to escalating debates on social media during the same time period. For the second wave of confirmed new cases, the major contributors, the South and West regions, did not ripple as many related tweets as the first wave. Our understanding is that the national level news attracted more attention and discussion all over the U.S. in the first wave, even though some regions were not impacted as much as the Northeast U.S.

We proposed and validated the feasibility of using Twitter users’ registration state as a proxy of tweeting location. This location approximation can be a helpful GIS solution for social media analysis, especially when the geotagged tweets are usually very limited. This framework can be extended to broader research projects which aim to use real-time and accessible tweets for geographical and temporal analysis. This framework can provide new and complementary perspectives promptly compared to data sources that need a long time to collect or conduct clinical experiments. In addition, the synchronized trend of tweets, new cases, and new deaths at the national level echoes past research (Lee, Agrawal, and Choudhary 2013; Liu and Young 2018), which suggested that Twitter could be used as a surveillance platform to monitor health-related topics such as disease, mobility, and more. We also observed that the second wave of confirmed cases did not ripple much in the Twitter community, reflecting the pandemic fatigue phenomenon. A possible explanation is that people were used to the pandemic norm or even became paralysed and powerless about the number of cases and deaths. This trend also mirrored the five-stage of grief (Kübler-Ross model), or a more recent adaption called the cycle of acceptance, which indicates most people go over denial, anger, depression, bargaining, and eventually reach acceptance after receipt of bad news (Kübler-Ross 1969; Mortazavi et al. 2020).

However, using social media for health communication during the pandemic has not always provided users with the best health information. A recent study by Kathleen Jamieson (Jamieson and Albarracín 2020) showed that social media provided the most misinformation concerning the pandemic than any other communication channel. The quality of health information provided is critical for those populations, like those with CVD, who are particularly vulnerable to COVID-19, and is important for disease prevention before the medical community realizes the link between CVD and COVID-19. While social media is a powerful and well-utilized channel for health communication, it appears to need some regulation to curb misinformation and improve health information dissemination. A recent study (Jamieson and Albarracín 2020) mentioned a similar idea that social media exposure correlates with a higher level of misinformation. The study further recommends social media platforms’ efforts to blunt misinformation from where it starts.

There are some limitations of our study. First, we used acronyms, such as CAD, to collect tweets. There may be multiple possibilities of the full names, such as coronary artery disease, which we are looking for, and computer-aided design, which is a skill or software. In this sense, there may be some unrelated tweets being collected. In our case, the percentage of unrelated tweets is very low since we used two sets of searching keywords for both COVID-19 and CVD. Second, as we limited the tweet to contain keywords from both COVID-19 and CVD, there were only 1,172 actual geotagged tweets. Although we justified using user registration location as the proxy and expanded the group of geotagged tweets, there is still some uncertainty in the data. The amount of collected data also constrained our analysis to the state level, or we will have a small sample size problem (Jones and Moon 1991). A more detailed analysis can be done using a big sample size of geotagged tweets. In addition, although Twitter provides precise GPS coordinates (if users opt in for the service), users can choose their geotag location subjectively. Due to the lack of ground truth, the challenges of validating contribute to data uncertainty. Third, we observed how information might be misinterpreted and disseminated among Twitter users, but we did not compare Twitter messaging with other social media platforms. Future research could analyse how health information is communicated, changed, and perceived over time and space via social media platforms. Scholars have done research understanding the relationship between COVID-19 dynamics with the environmental and demographic characteristics of different places, including Italy, Spain, and the U.S. (Coccia 2020; Oto-Peralías 2020; Abolfazl, Vahedi, and Rivera 2020). It will be interesting to extend the correlation analysis to include socioeconomic status, and health measures, such as ICU availability, disease prevalence, vulnerable population proportion, and more. Due to data limitations, we only presented the temporal variation of COVID-19 and CVD related tweets at the state level, and the spatial variation between tweets, cases, and deaths at the region level. In future work, we are interested in expanding the analyses to more geographic and temporal scales with the acquisition of better data.

## Figures and Tables

**Table 1.** Tweets searching keywords.

Literature		CVD keywords	COVID-19 Keywords
CVD		CVD	COVID
		Heart disease	Coronavirus
		Cardiovascular disease	SARS-COV-2
Top five cardiac diagnoses on ICU admission	CAD & AMI (Coronary artery disease & acute myocardial infarction)	CAD	
		Coronary artery disease	
		AMI	
		Heart attack (symptom)	
		Myocardial infarction	
		Acute MI	
	Congestive heart failure	Heart failure (alternative name)	
	Rhythm disturbance	Rhythm disturbance	
		Arrhythmias (alternative name)	
	Cardiogenic shock	Cardiogenic shock	
	Trauma	Cardiac injury	
		Heart injury	
		Chest trauma	
		Cardiac trauma	

**Table 2.** Correlation coefficients between smoothed tweet count, new cases, and new deaths of COVID-19 in the U.S.

Correlation coefficient	Smoothed tweet count	Smoothed new cases	Smoothed new deaths
Smoothed tweet count	1		
Smoothed new cases	0.63	1	
Smoothed new deaths	**0.88**	0.65	1
